# Does taking endurance into account improve the prediction of weaning outcome in mechanically ventilated children?

**DOI:** 10.1186/cc3898

**Published:** 2005-11-16

**Authors:** Odile Noizet, Francis Leclerc, Ahmed Sadik, Bruno Grandbastien, Yvon Riou, Aimée Dorkenoo, Catherine Fourier, Robin Cremer, Stephane Leteurtre

**Affiliations:** 1 Paediatric Intensive Care Unit, University Hospital of Lille, Rue Eugène Avinée, 59037 Lille Cedex, France; 2 Department of Epidemiology, University Hospital of Lille, Rue Eugène Avinée, 59037 Lille Cedex, France; 3 Department of Respiratory Physiology, University Hospital of Lille, Rue Eugène Avinée, 59037 Lille Cedex, France

## Abstract

**Introduction:**

We conducted the present study to determine whether a combination of the mechanical ventilation weaning predictors proposed by the collective Task Force of the American College of Chest Physicians (TF) and weaning endurance indices enhance prediction of weaning success.

**Method:**

Conducted in a tertiary paediatric intensive care unit at a university hospital, this prospective study included 54 children receiving mechanical ventilation (≥6 hours) who underwent 57 episodes of weaning. We calculated the indices proposed by the TF (spontaneous respiratory rate, paediatric rapid shallow breathing, rapid shallow breathing occlusion pressure [ROP] and maximal inspiratory pressure during an occlusion test [Pi_max_]) and weaning endurance indices (pressure-time index, tension-time index obtained from P_0.1 _[TTI_1_] and from airway pressure [TTI_2_]) during spontaneous breathing. Performances of each TF index and combinations of them were calculated, and the best single index and combination were identified. Weaning endurance parameters (TTI_1 _and TTI_2_) were calculated and the best index was determined using a logistic regression model. Regression coefficients were estimated using the maximum likelihood ratio (LR) method. Hosmer–Lemeshow test was used to estimate goodness-of-fit of the model. An equation was constructed to predict weaning success. Finally, we calculated the performances of combinations of best TF indices and best endurance index.

**Results:**

The best single TF index was ROP, the best TF combination was represented by the expression (0.66 × ROP) + (0.34 × Pi_max_), and the best endurance index was the TTI_2_, although their performance was poor. The best model resulting from the combination of these indices was defined by the following expression: (0.6 × ROP) – (0.1 × Pi_max_) + (0.5 × TTI_2_). This integrated index was a good weaning predictor (*P *< 0.01), with a LR^+ ^of 6.4 and LR^+^/LR^- ^ratio of 12.5. However, at a threshold value <1.3 it was only predictive of weaning success (LR^- ^= 0.5).

**Conclusion:**

The proposed combined index, incorporating endurance, was of modest value in predicting weaning outcome. This is the first report of the value of endurance parameters in predicting weaning success in children. Currently, clinical judgement associated with spontaneous breathing trials apparently remain superior.

## Introduction

Weaning (or discontinuation) from mechanical ventilation is definitive cessation of mechanical ventilation and differs from extubation, which is removal of the endotracheal tube [1,2]. Determining the optimal time at which to discontinue mechanical ventilation must not be based simply on clinical impression because weaning depends on multiple factors [2,3]: central drive and peripheral nerves; mechanical respiratory loads, ventilatory muscle properties and gas exchange properties; and cardiac tolerance and peripheral oxygen demands. Premature weaning places the patient at risk for reintubation and airway trauma, whereas delayed weaning exposes them to risk for nosocomial infection and increases hospitalization costs. Indeed, 'the complexity of the decision to extubate provides a strong rationale for developing accurate predictors of extubation outcome' [3].

Extensive efforts have been made to identify predictors of successful weaning in adults [4,5] as well as in children [6-25]. The following indices were proposed by the Collective Task Force of the American College of Chest Physicians (TF) as the most promising weaning predictors [5]: spontaneous respiratory rate, paediatric rapid shallow breathing (RSB) [13], RSB occlusion pressure (ROP) [26] and maximal inspiratory pressure during an occlusion test (Pi_max_) [23,27]. Although none of these predictors appears to be sufficiently sensitive or specific in predicting weaning success, paediatric studies have used integrated indices, including respiratory drive, respiratory load, muscle strength and quality of gas exchange.

Fatigue (for example, diminution of endurance) of the inspiratory muscles is defined as reduction in capacity to develop force and/or velocity of a muscle, which results from muscle activity under load and is reversible with rest [2,28-30]. The two reference techniques for assessing endurance (for example, detecting fatigue) are analysis of the change in the electromyographic power spectrum and in the force response of muscles to electrical stimulation [2]. Another approach uses the tension-time index (TTI) of the diaphragm, calculated from the mean transdiaphragmatic pressure, which correlates well with reference techniques. However, because determination of transdiaphragmatic pressure is invasive, noninvasive TTI estimated from the measurement of mouth occlusion pressure (P_0.1_) and from the integral of the airway pressure curve over time during spontaneous ventilation were used as predictors of weaning success [2]. Finally, Jabour and coworkers evaluated ventilatory endurance using a modified TTI, namely the pressure-time index (PTI), which was calculated from peak airway pressure during mechanical ventilation [28].

Respiratory muscle endurance, which has been reported to have particular significance in predicting weaning success in adults [28-30], has never been investigated in children. Thus, the aims of the present prospective study were as follows: to evaluate the ability of indices proposed by the TF [5] and endurance indices to predict weaning outcome; and to determine whether a combined index, including the most accurate TF and endurance indices, could enhance the ability to predict weaning success.

## Materials and methods

This prospective study was approved by the local hospital institutional review board, and parents provided informed consent before the study began. All children admitted to the paediatric intensive care unit at our university-affiliated hospital from March 1999 to July 2001, and who required mechanical ventilation for more than 6 hours were eligible for inclusion in the study. Children with chronic neuromuscular disease, who had undergone tracheostomy, or who were aged under 30 days were excluded. The primary physician was responsible for the weaning decision and process, and was blinded to the results of the measurements performed during the short period of spontaneous breathing (see below). All ventilatory variables were collected by the same investigator, and the weaning procedures were not delayed in his absence (for example nights and weekends); those patients who did undergo weaning procedures in his absence were excluded from the study.

Patients were enrolled in the study if they met all the following criteria, as defined for adults by the French Society of Critical Care Consensus Conference [31]: improvement or resolution of the underlying cause of acute respiratory failure; core temperature <38.5°C; satisfactory renal function; no signs of infection; neuropsychological state compatible with autonomous breathing; correction of electrolyte disorders; a haemoglobin level above 9 g/dl; absence of left ventricular dysfunction or cardiac arrhythmia; no sedation or mild sedation (for example, midazolam <0.5 μg/kg per minute, morphine sulphate <1 mg/kg per day, or fentanyl citrate <0.25 μg/kg per hour); efficient cough; and adequate gas exchange. Moreover, all of the patients were weaned using pressure support ventilation (PSV) on Servo 300 or Servo 900C ventilators (Siemens-Elema, Solna, Sweden), with a pressure support below 15 cmH_2_O, a positive end-expiratory pressure below 5 cmH_2_O and fractional inspired oxygen (FiO_2_) below 40% [31]. After the patient's primary physician made the decision to wean, the patient's respiratory variables were recorded by the investigator, first during mechanical ventilation and then during a short period of spontaneous breathing. Then, the children underwent a spontaneous breathing trial (SBT) through a T-piece circuit or Canopy device for 30 minutes [32]. Then, children were extubated, depending on their clinical status and blood gas determination [31]. Patients who failed SBT were excluded. Weaning failure was defined as reinstitution of invasive or noninvasive mechanical ventilation within 48 hours of extubation, for a reason than upper airway obstruction.

### Measurements

Demographic data collected included age, weight, sex, admission diagnosis, Paediatric Risk of Mortality (PRISM) score calculated during a 24-hour period of observation, tube internal diameter and duration of ventilation. Ventilator settings, recorded when the child was under PSV, included ventilator rate (for example, respiratory rate [RR]_PSV_), peak inspiratory pressure, positive end-expiratory pressure, mean airway pressure, tidal volume (VT) and FiO_2_. Then, during a short period of spontaneous breathing, respiratory parameters were measured using a calibrated Fleish no. 0 pneumotachograph (MSR, Paris, France) connected to a ± 2 cmH_2_O differential pressure transducer (Validyne, Northridge, CA, USA) over 15–30 consecutive breaths. Measured parameters included spontaneous respiratory rate (RR_s_), spontaneous VT (for example, VT_s_), inspiratory time (T_i_) and total respiratory cycle time (T_tot_). Pi_max _was measured by occluding the airway for a least 20 s using a unidirectional valve system (LSA, Paris, France) that allowed expiration but not inspiration, and a ± 50 cmH_2_O differential pressure transducer (Validyne). Initial inspiratory pressure was measured as the negative pressure deflection produced by the first inspiration attempt, whereas Pi_max _was taken as the most negative deflection produced by any inspiration attempt during airway occlusion [9,23,27]. Negative pressure 0.1 s after airway occlusion (P_0.1_) was measured by occluding the airways at the end-expiratory level, with a vibration- and noise-free pneumatic valve (Hans-Rudolph, Kansas City, KS, USA) [8,33].

Measurements were performed by the same investigator and were repeated three times; mean values were used for data analysis. Between each occlusion trial, sufficient time was allowed to ensure that the patient's arterial oxygen saturation and heart rate had returned to their previous baseline values. VT_s _and VT_PSV _were corrected for body weight.

Respiratory parameters calculated included duration of mechanical ventilation, arterial oxygen tension/FiO_2 _ratio [34,35], paediatric RSB [13], PTI [28,36], ROP [26], TTI obtained from P_0.1 _(TTI_1_) [37,38] and TTI obtained from mean airway pressure (TTI_2_) [39]. Formulae are summarized in Table 1. Age-adjusted RR was calculated using a Z score [14,19,20,40] and used for paediatric RSB and ROP calculations. Adjustment to age was done for P_0.1 _and age-adjusted values were used for TTI and ROP calculations [41].

### Statistical analysis

The distribution of data was expressed as medians with 25th and 75th percentile ranges (Q1–Q3). Comparison of continuous variables between the two outcome groups (weaning success and weaning failure) was done using Kruskal-Wallis test. A χ^2 ^test or Fisher's exact test, when expected number was less than 3, were used for comparison of categorical variables between two groups. All if the indices, including RR and P_0.1_, were studied before and after age adjustment.

Sensitivity, specificity, positive predictive values and negative predictive values (NPVs) were calculated using standard formulae. A true positive/negative result was defined as occurring when a test predicted weaning success/failure and weaning actually succeeded/failed. A false-positive result occurred when a test predicted success but weaning failed, and a false-negative result was when a test predicted weaning failure but weaning succeeded [34,35]. General performance of each index was assessed using positive and negative likelihood ratios (LR^+ ^and LR^-^, respectively) [2,42-45], calculated for each index [46] after discretization in dichotomous variables. An index could be predictive (LR^+ ^>2, LR^- ^<0.5, or LR^+^/LR^- ^ratio >4), well predictive (LR^+ ^>5, LR^- ^<0.2, or LR^+^/LR^- ^ratio >10), or very well predictive (LR^+ ^>10, LR^- ^<0.1, or LR^+^/LR^- ^ratio >100) [4,42,43].

The performances of each individual TF index and combinations of them were calculated, and the best performers were identified from among the individual TF indices and TF combinations. In the same manner, the performances of reported weaning indices, including endurance parameters (TTI_1 _and TTI_2_), were calculated and the best endurance index was identified. All variables significant at the *P *< 0.20 level in the univariate analysis were included in a stepwise logistic regression model using a *P*-to-remove at 0.05. Regression coefficients were estimated using the maximum likelihood method. The Hosmer–Lemeshow test was used to estimate goodness-of-fit of the model [47]. A final equation was constructed to predict weaning success. Finally, the performance of combinations of best TF indices and best endurance index was calculated.

Area under the receiver operating characteristic curves (AUC) was calculated using the nonparametric method proposed by Hanley and McNeil [48], and the 95% confidence interval was calculated using the Wilcoxon test. Results could be considered uninformative (AUC = 0.5), poorly accurate (0.5 < AUC = 0.7), moderately accurate (0.7 < AUC = 0.9), highly accurate (0.9 < AUC = 1), or perfect (AUC = 1) [49,50]. *P *< 0.05 was considered statistically significant. In summary, the AUC, Hosmer–emeshow goodness-of-fit test, LR test, and well predicted outcome percentages were used to evaluate predictive accuracy.

## Results

Of the 220 patients who were eligible for inclusion in the study, 56 patients were enrolled (25%) and underwent 59 episodes of weaning (median age 29 [11–94] months, median weight 13 [9-23] kg). The median duration of ventilation was 4 (2–9) days, and 39 patients received mild sedation during the weaning procedure. Two patients who did not tolerate SBT were excluded. Of the remaining 54 children, 17 patients had neurologic disorders (30%), 28 had respiratory failure (49%), three had both neurologic disorders and respiratory failure (5%) and nine underwent surgery (16%). Of the remaining 57 episodes of weaning (in the 54 children), 45 attempts were successful (80%) and 12 were unsuccessful (20%; Table 2). The causes of reintubation were respiratory failure (*n *= 6; one child with spinal amyotrophy, one with myopathy and one with mucopolysaccharidosis), pulmonary oedema of cardiac origin (*n *= 3), bronchial obstruction (*n *= 2; 1 burned child) and atelectasis (*n *= 1). Three of the 12 patients who failed weaning needed noninvasive ventilation within 48 hours after extubation (20%) and nine required reintubation and mechanical ventilation for a reason other than upper airway obstruction (80%). Two patients who were successfully extubated required reintubation for severe upper airway obstruction (4%). Age, sex, weight, PRISM score, duration of ventilation and internal tube diameter were not statistically different between the weaning success and weaning failure groups (Table 3). Also, diagnoses were not statistically different between the groups. Finally, ventilator settings and spontaneous breathing parameters at the beginning of the weaning procedure were not significantly different between the two groups (Table 4).

The best single TF index was ROP, the best combination of TF indices was represented by the expression (0.66 × ROP) + (0.34 × Pi_max_), and the best endurance index was the TTI_2_. However, their performance was statistically poor (Table 5). The best model resulting from the combination of these indices was defined by the expression: (0.6 × ROP) – (0.1 × Pi_max_) + (0.5 × TTI_2_). This integrated index fitted the data well (*P *= 0.364, Hosmer–Lemeshow test) and was a good weaning predictor (*P *< 0.01, Kruskal-Wallis test). The threshold value that best discriminated between two groups was 1.3, with a value <1.3 predicting weaning success (*P *= 0.007, Fisher test). Although LR^+ ^was 6.4 and the LR^+^/LR^- ^ratio was greater than 10, LR^- ^remained above 0.2, meaning that this integrated index was only predictive of weaning success.

## Discussion

In a population of 54 critically ill children, we found that the indices proposed by the TF were insufficient in predicting weaning outcome. Among the combined indices, including endurance in order to enhance prediction of weaning outcome, the best combination was defined by the expression: (0.6 × ROP) – (0.1 × Pi_max_) + (0.5 × TTI_2_). However, this index was not a valuable predictor of weaning outcome because it only predicted weaning success.

The best combination of TF indices included ROP and Pi_max_. Although Pi_max _was considered by the TF [5] to be one of the most promising weaning predictors in adults (based on LR^+ ^and LR^-^), no data concerning LRs were available for ROP in the adult or paediatric literature [26]. In the present study ROP alone was only predictive of weaning success, and this finding is in agreement with that obtained in a population of 45 adult patients by Sassoon and Mahutte [26], who found that ROP may be useful and more accurate in predicting weaning outcome when ROP components (P_0.1 _and RSB) alone were indeterminate [26]. The AUC (0.80 ± 0.9) and LRs (LR^+ ^2.42, LR^- ^0.05, and LR^+^/LR^- ^48), recalculated from their published data, appear to be better than those in our study. Pi_max_, which is considered a global index of respiratory muscle strength [35,38,51,52] and was adapted to children by El-Khatib and coworkers [23], was evaluated in five paediatric studies [6,8-10,12], but LRs were calculated in only one study designed to predict weaning failure [12]. The LRs were recalculated to predict weaning success, and the results (LR^+ ^1.09, LR^-^0.35, and LR^+^/LR^-^3.11) appeared similar to those obtained in the present study (Tables 5 and 6) and in five adult studies [5]. AUC values obtained in the five paediatric studies were similar to those in our study and ranged from 0.53 (95% confidence interval 0.47–0.67) [8] to 0.57 (95% confidence interval 0.47–0.67) [12]. However, in a recent adult study that included 52 patients [50], Pi_max _was a good weaning predictor (LR^+ ^1.40, LR^- ^0.12, and LR^+^/LR^- ^11.67).

In our study, TTI_2 _was found to be the best endurance index, although it was not very predictive of weaning success (Table 5). Numerous studies indicate that duration of inspiration relative to the duration of the total respiratory cycle (for example, T_i_/T_tot _ratio) should be an important determinant of diaphragmatic fatigue [35,53-55]. Hence, any increase of indices that include T_i_/T_tot _ratio such as PTI [28,36], TTI_1 _[37,38] and TTI_2 _[39] could reflect decreased endurance. In adults, Ramonaxto and coworkers [56] reported that TTI_2 _highly correlated with TTI of the diaphragm, which was proposed by Bellemare and Grassino [54,55] for quantifying the magnitude and duration of respiratory muscle contraction. However, TTI_2 _has never been used as a predictor of weaning outcome either in adults or children, whereas TTI_1 _and PTI appeared to be useful predictors of weaning outcome in adults [28,56].

Several adult and paediatric studies [7,9,13-15] have attempted to define integrated weaning indices that include parameters in relation to respiratory muscle function, such as respiratory drive, respiratory load and muscle strength [35,53,57], but none of these integrated indices was found to be predictive of weaning outcome. Respiratory muscle endurance has not been investigated in paediatric patients; however, the diaphragm is less endurant in children than in adults because of anatomic and physiologic differences [58]. Furthermore, in adults respiratory muscle endurance was reported to be of particular significance in predicting weaning outcome [28-30]. Taking respiratory muscle endurance into account should improve prediction of weaning success. In the present study TTI_2 _was found to contribute strongly to the integrated index, and its association with ROP and Pi_max _improved LR^+^, LR^- ^and LR^+^/LR^- ^ratio.

Comparing the performance of weaning indices is difficult because few studies have calculated LRs, which are considered as the best tests [4]. Indeed, only one paediatric study calculated LRs [12], and most other paediatric studies did not report the number of true-positive and false-positive findings. Furthermore, large discrepancies exist in the literature regarding population characteristics, the definition of weaning and extubation [59], inclusion and exclusion criteria (for example aetiology of respiratory failure and duration of ventilation), the definition of weaning success and weaning failure, and the use (or not) of noninvasive mechanical ventilation after extubation [16,23].

Three factors may explain why integrated indices do not improve upon the accuracy of prediction of weaning outcome of single indices. First, most paediatric studies included small numbers of patients with a relative low rate of weaning failure, and it has been demonstrated that a large sample including more than 1,000 patients with at least 100 extubation (or weaning) failures would be necessary to assess adequately the ability of parameters to predict extubation (or weaning) success in children [60]. Second, weaning indices are often measured too late, when patients meet all of the clinical criteria for weaning, and so threshold values are not very discriminatory [5]. Third, SBT is considered to be the most direct and simplest way to assess patient performance without ventilator support [2], and so a failed SBT is synonymous of weaning failure. Thus, the number of false-negative findings (for example, patients who failed SBT but who could have been weaned) is unknown, and the specificity, NPV and LR^- ^of SBT cannot be determined [2]. Among 105 extubations in adults considered to be false negative because of a pre-extubation respiratory rate above 30 breaths/minute, DeHaven and coworkers [17] observed that 97 were successful. Furthermore, Epstein and coworkers [3] noted that 30–70% of unplanned extubation in adult patients did not result in reintubation.

Weaning is usually delayed by clinicians [3], and a weaning index should be more predictive of weaning failure (for example, it should be more specific, and have a good NPV and a lower LR^-^). Thus far, all paediatric studies of weaning indices, which determined their sensitivity, specificity, LRs, or AUCs, included a SBT. As long as a SBT is defined as a necessary pre-condition for extubation by the TF [2], comparisons between SBT and weaning indices will be limited because of the underestimation of false-negative findings and NPV.

Our study has two other limitations. First, there was no defined protocol for decreasing mechanical ventilation, although there is no evidence that such a protocol must be used in children [4,61]. Moreover, we employed no set protocol for decreasing sedation, which is supposed to improve weaning success in children [59]. Second, during the study period 220 patients were eligible according to the inclusion criteria, but only 27% of these were included. Like the study conducted by Thiagarajan and coworkers [14], in which 227 children from among 472 admitted to their paediatric intensive care unit (48%) were included, our study design required the presence onsite of the investigator in charge of data collection in order to decrease variability in measurement of the different parameters and to guarantee that the patient's physician remained blinded to the results.

The weaning failure rate was 20%, which is similar to that reported by others in paediatric patients, ranging from 1.4% [62] to 34% [12]. The median PRISM value, calculated within 24 hours of admission, was close to that reported in most previous paediatric studies [11,12,16,63], although PRISM values were found to be lower in two studies [62,64].

## Conclusion

In our population of 54 critically ill children, indices proposed by the TF were insufficient in predicting weaning outcome. The best combined index, incorporating endurance, was defined by the expression: (0.6 × ROP) – (0.1 × Pi_max_) + (0.5 × TTI_2_). This is the first report of the value of endurance parameters in predicting weaning success in children. The index had a LR^+ ^of 6.4 and a LR^+^/LR^- ^ratio above 10; however, at a threshold value <1.3 it was only predictive of weaning success (LR^- ^>0.2). Although this index cannot be considered sufficient for making decisions regarding weaning, the reason for this is probably that it was measured, like in other studies, when children met all clinical criteria for weaning. At the present time, clinical judgement associated with SBT still seems superior in the weaning process in children.

## Key messages

• 	Determining the optimal time to discontinue mechanical ventilation is important, because premature weaning places the patient at risk for reintubation and delayed weaning exposes the patient to risk for nosocomial infection.

• 	The indices proposed by the TF are not sufficiently sensitive or specific in predicting weaning outcome.

• 	Until now, indices that reflect respiratory muscle endurance have not been investigated in children.

• 	Our best index, combining ROP, Pi_max _and TTI_2_, was only predictive of weaning success.

• 	Such indices are unable to assist with decisions regarding weaning probably because they are measured when patients meet all clinical criteria for weaning.

## Abbreviations

AUC = area under the curve; FiO_2 _= fractional inspired oxygen; LR = likelihood ratio; NPV = negative predictive value; Pi_max _= maximal inspiratory pressure during an occlusion test; PRISM = Paediatric Risk of Mortality Score; PSV = pressure support ventilation; PTI = pressure-time index; ROP = RSB occlusion pressure; RR = respiratory rate; RSB = rapid shallow breathing; SBT = spontaneous breathing trial; TF = Task Force of the American College of Chest Physicians; T_i _= inspiratory time; TTI = tension-time index; T_tot _= total respiratory cycle time.

## Competing interests

The authors declare that they have no competing interests.

## Authors' contributions

BG performed the statistical analysis and interpretation of the data. ON, FL, BG, YR conceived the study, participated in the design and execution of the study, the analysis of data and writing of the manuscript. BG performed the statistical analysis and interpretation of the data. AS performed respiratory investigations. ON, AD, CF, RC, SL performed clinical data collection. All authors read and approved the final manuscript.

## Figures and Tables

**Table 1:** Index formulas

Index [reference]	Definition	Formula
RSB_p _[13]	Paediatric rapid shallow breathing	RR_s_/VT_s_
ROP [26]	Rapid shallow breathing occlusion pressure	= P_0.1 _× (RR_s_/VT_s_)
PTI [28]^a,b^	Pressure–time index	= (P_breath_/Pi_max_) × (T_i_/T_tot_) P_breath _= (PIP - PEP)/(VT_s_/VT_PSV_)
TTI_1 _[37,38]	Tension–time index 1	= (P_imoy_/Pi_max_) × (T_i_/T_tot_) P_imoy _= 0.5 × (P_0.1 _× 10) × T_i_
TTI_2 _[39]	Tension–time index 2	= (P_aw_/Pi_max_) × (T_i_/T_tot_)

^a^Paediatric literature; ^b^adult literature. P_0.1_, negative pressure 0.1 s after airway occlusion (cmH_2_O); P_aw_, mean airway pressure on pressure support ventilation (cmH_2_O); P_breath_, pressure generated by unassisted breath (cmH_2_O); PEEP, positive end-expiratory pressure on pressure support ventilation (cmH_2_O); PIP, peak inspiratory pressure on pressure support ventilation (cmH_2_O); Pi_max_, maximal inspiratory pressure during occlusion test (cmH_2_O); P_imoy_, spontaneous mean inspiratory airway pressure (cmH_2_O); PTI, pressure–time index (no unit); ROP, rapid shallow breathing occlusion pressure (cmH_2_O/breaths per min per ml); RR_s_, spontaneous respiratory rate (breaths/min); RSBp, paediatric rapid shallow breathing (breaths/min per ml per kg); T_i_, inspiratory time (s); T_i_/T_tot_, inspiratory duty cycle; TTI_1_, tension–time index obtained from the P_0.1 _value (%); TTI_2_, tension–time index obtained from the P_aw _value (%); VT_PSV_, tidal volume on pressure support ventilation (ml/kg); VT_s_, spontaneous tidal volume (ml/kg).

**Table 2:** Characteristics of children enrolled in the study

Patient (number)	Weight (kg)	Age (months)	Group	PRISM	Ventilation duration (hours)	Diagnosis
Weaning success						
1	4.4	1.5	2	13	156	Intracranial haemorrhage
2	32.5	137	1	13	44	Meningococcal septic shock
3	6.2	7	2	12	266	Intracranial haemorrhage
4^a^	9.8	10	1	10	216	ARDS
5^a^	8.7	11	1	10	391	ARDS
6	18.5	75	3	11	32	Postoperative state
7	10.9	14	2	12	263	Traumatic brain injury
8	5	3	2	11	270	Traumatic brain injury
9	35	134	2	17	142	Encephalitis
10	3.7	1.5	1	29	213	Acute cardiac failure
11	12.2	27	1	6	36	Pneumonia
12	54	169	1	8	44	Asthma
13	7.07	11	1	0	674	Myocardial infarction
14	11	19	2	4	41	Metabolic brain damage
15^b^	16.2	29	1	19	214	Meningococcal septic shock
16	16.6	65	1	4	54	Pneumonia
17	5.5	3	2	4	176	Intracranial haemorrhage
18	10.5	22	1	0	79	Asthma
19	12.2	18	2	10	74	Traumatic brain injury
20	12	24	2	13	38	Traumatic brain injury
21	47	165	1	13	112	Asthma
22	10.5	18	4	1	88	Pneumonia, brain injury
23	12	12	2	22	87	Pneumonia
24	5.5	3	2	10	221	Intracranial haemorrhage
25	37	93	2	0	53	C3–C4 cervical injury
26	4.3	4	1	13	93	Pneumonia
27	17	85	2	22	170	Rhombencephalitis
28	10	21	1	5	14	Pneumonia
29	24	123	3	6	24	Postoperative state
30	21	94	3	6	26	Postoperative state
31	25.6	103	1	19	75	Asthma
32	25	97	3	4	25	Postoperative state
33	20	88	1	9	20	Myopathy, pneumonia
34	12.5	30	4	6	20	Brain injury, Pneumonia
35	10	14.5	3	10	28	Postoperative state
36^c^	7.5	6.5	2	15	167	Burkitt's lymphoma
37	17.1	74	2	8	96	Encephalitis
38	7	10.5	3	6	27	Postoperative state
39	36	123	1	3	500	Acute heart failure
40	20	76	2	21	210	Encephalitis
41	11.7	33	3	7	13	Postoperative state
42	3.2	1	3	4	342	Postoperative state
43	7.2	9	2	0	129	Encephalitis
44	30	119	3	16	49	Postoperative state
45	54.9	184	1	11	541	ARDS
Weaning failure						
46	15	33	2	14	193	Encephalitis
47	20	60	1	19	44	Carbon dioxide poisoning
48	8.6	8	1	22	533	Meningococcal septic shock
49	4.6	3	1	20	43	Acute cardiac failure
50	56.8	187	1	3	72	Pneumonia,
51	16.6	51	4	16	138	Traumatic brain injury, lung injury
52^b^	16	29	1	19	180	Meningococcal septic shock
53	18	152	1	12	128	ARDS
54	5.6	6	1	4	53	Pneumonia
55	8.9	17	1	15	101	Myocardiopathy
56	54	129	1	1	216	Pneumonia, myopathy
57^c^	36	123	1	3	357	Burkitt's lymphoma
Exclusion						
58	13	36	2	15	45	Toxic brain injury
59	25	80	2	5	587	Medullary tumor

Diagnoses were classified into four groups: group 1, respiratory failure; group 2, neurological disorder; group 3, postoperative state; and group 4, respiratory failure and neurological disorder. Three patients were included twice in the study (^a^, ^b ^and ^c^). ARDS, acute respiratory distress syndrome.

**Table 3:** Characteristics of the 54 children who underwent 57 episodes of weaning

Variable	Weaning success (*n *= 45)	Weaning failure (*n *= 12)	*P*
Age (months)	24 (10–93)	43 (20–112)	NS
Sex (male/female)	32/13	10/2	NS
Weight (kg)	12 (7–23)	16 (10–24)	NS
PRISM score	10 (5–13)	15 (4–18)	NS
Duration of ventilation (days)	4 (2–9)	6 (2–9)	NS
Tube internal diameter (mm)	4.5 (4.0–5.0)	5 (4.0–6.5)	NS
Sedation before weaning procedure (*n*)	30	9	NS

PRISM score was calculated after a 24-hour period. Values are expressed as median (25th and 75th percentile range). NS, not significant; PRISM, pediatric risk of mortality.

**Table 4:** Preweaning ventilator settings and spontaneous breathing parameters

Variable	Weaning success	Weaning failure	*P*
PIP (cmH_2_O)	14 (12–16)	15 (14–16)	NS
VT_PSV _(ml/kg)	8.9 (7.7–10.1)	8.9 (6.1–10.1)	NS
RR_s _not age-adjusted (breaths/minute)	40 (30–57)	59 (47–67)	NS
RR_s _age-adjusted (breaths/minute; ref.)	2.4 (0.8–6.3)	7.9 (3.2–11.4)	NS
VT_s _(ml/kg)	7.1 (5.7–9.8)	7.1 (4.5–8.9)	NS
PaCO_2 _(mmHg)	33 (30–38)	35 (32–36)	NS
PaO_2 _(mmHg)	78 (65–87)	69 (64–76)	NS
FiO_2 _(%)	0.25 (0.21–0.30)	0.30 (0.24–0.36)	NS
PaO_2_/FiO_2 _(mmHg)	296 (247–371)	237 (190–310)	NS

FiO_2_, inspired oxygen fraction; NS, not significant; PaCO_2_: arterial carbon dioxide partial pressure; PaO_2_, arterial oxygen partial pressure; PIP, peak inspiratory pressure on pressure support ventilation; RR_s_, spontaneous respiratory rate; VT_PSV_, tidal volume on pressure support ventilation; VT_s_, spontaneous tidal volume.

**Table 5:** Accuracy of the separate and combined weaning indices

Index/combination	Threshold value^a^	LR^+^	LR^-^	LR^+^/LR^- ^ratio	PPV	NPV	AUC (95% CI)
Single TF indices^b^							
RR_s _(breaths/minute)	< 5	1.69	0.31	5.45	0.86	0.46	0.72 (0.55–0.89)
RSBp (breaths/minute per ml per kg)	< 2	1.21	0.36	3.36	0.82.	0.43	0.71 (0.56–0.87)
*ROP (cmH_2_O/breaths per minute per ml)*	<*10*	*2.13*	*0.43*	*4.73*	*0.89*	*0.38*	*0.72 (0.55–0.88)*
*Pi_max _(cmH_2_O)*	> *50*	*1.38*	*0.62*	*2.23*	*0.84*	*0.30*	*0.56 (0.35–0.77)*
Endurance indices							
PTI (no unit)	< 0.08	1.47	0.53	2.77	0.85	0.33	0.61 (0.43–0.79)
TTI_1 _(%)	< 0.2	1.53	0.73	2.10	0.85	0.27	0.55 (0.36–0.74)
*TTI_2 _(%)*	<*0.05*	*1.71*	*0.50*	*3.42*	*0.86*	*0.35*	*0.65 (0.47–0.84)*
TF combinations^c^							
(0.66 × RR_s_) + (0.34 × Pi_max_)	< 1.3	2.49	0.50	4.98	0.90	0.35	0.72 (0.55–0.89)
(0.5 × RSB) + 0.5 Pi_max_	< 1.5	1.60	0.57	2.81	0.86	0.32	0.63 (0.45–0.82)
*(0.66 × ROP) *+ *(0.34 × Pi_max_)*	<*1.5*	*2.13*	*0.43*	*4.95*	*0.89*	*0.38*	*0.74 (0.60–0.89)*
Best TF^d ^and best endurance index combination							
(0.6 × ROP) – (0.1 × Pi_max_) + (0.5 × TTI_2_)	< 1.3	6.4	0.51	12.55	0.96	0.34	0.76 (0.62–0.90)

All parameters, including RRs, are adjusted to age by a Z-score. All parameters, including P_0.1_, are adjusted to age, in accordance with the literature. ^a^Best predicting weaning success. ^b^Performance of each index recommended by the TF. ^c^Combinations of single TF indices; italic text indicates the best performing combination. ^d^Best index from among single TF indices and TF combinations. AUC, area under the receiver operating characteristic curve; CI, confidence interval; LR^+^, positive likelihood ratio; LR^-^, negative likelihood ratio; NPV, negative predictive value; P_imax_, maximal inspiratory pressure during an occlusion test; PPV, positive predictive value; PTI, pressure-time index; ROP, rapid shallow breathing occlusion pressure; RRs, spontaneous respiratory rate; RSBp, paediatric rapid shallow breathing; TF, Task Force facilitated by the American College of Chest Physician; TTI_1_, tension-time index obtained from the P_0.1 _value; TTI_2_, tension-time index obtained from the P_aw _value.

**Table 6:** Performance of weaning indices recommended by the TF analysis

Index	Data	Summary LR^+ ^values	Summary LR^- ^values	Summary LR^+^/LR^- ^values	Threshold value
RRs	Binary	2.23	0.09	24.78	>30–38 breaths/minute
	Continuous	1.11	0.23	4.83	>38 breaths/minute
RSB	Binary	1.66	0.11	15.09	>100 breaths/minute per l
	Continuous	2.10	0.11	19.09	>100 breaths/minute per l
ROP	Binary	No data	No data	No data	<450
	Continuous	No data	No data	No data	
Pi_max_	Binary	1.32	0.31	4.26	<20 cmH_2_O
	Continuous	1.15	0.50	2.30	<20 cmH_2_O

Summarized is the performance of weaning indices recommended by the TF analysis [5]; no study was performed in pediatric patients. LR^+^, positive likelihood ratio; LR^-^, negative likelihood ratio; Pi_max_, maximal inspiratory pressure dring an occlusion test; ROP, rapid shallow breathing occlusion pressure; RRs, spontaneous respiratory rate; RSBp, paediatric rapid shallow breathing; TF, Task Force of the American College of Chest Physicians.
